# Duration and Risk Factors of Post-COVID Symptoms Following Recovery Among the Medical Doctors in Bangladesh

**DOI:** 10.7759/cureus.15351

**Published:** 2021-05-31

**Authors:** Sarmin Sultana, Mohammad Tanvir Islam, Marium Salwa, Shah M Zakir Hossain, Md Nazmul Hasan, Abdullah A Masum, Abed H Khan, Md Maruf Haque Khan, M Atiqul Haque

**Affiliations:** 1 Department of Public Health and Informatics, Bangabandhu Sheikh Mujib Medical University, Dhaka, BGD; 2 Department of Internal Medicine, Bangabandhu Sheikh Mujib Medical University, Dhaka, BGD; 3 Department of Nephrology, Bangabandhu Sheikh Mujib Medical University, Dhaka, BGD

**Keywords:** covid-19, medical doctors, duration, risk factors, post-covid symptoms

## Abstract

A large number of coronavirus disease 2019 (COVID-19) recovered patients are suffering from related symptoms. We conducted telephone interviews with 186 COVID-19 recovered medical doctors to determine the post-COVID symptoms, duration, and associated risk factors. About 70% of participants had at least one acute post-COVID symptom, including fatigue (43.0%), sleep disturbance (13.4%), lack of concentration (11.8%), breathing difficulty (10.2%), headache (6.5%), and muscle pain (6.5%). However, about 24% of participants reported having long post-COVID symptoms. Logistic regression analysis showed that female sex (odds ratio {OR}, 2.79; 95% CI, 1.28-6.06; p-value: 0.010) and comorbid conditions (OR, 2.28; 95% CI, 1.08-4.79; p: value, 0.030) are risk factors for the long post-COVID symptoms.

## Introduction

Around 131 million people have been infected worldwide since the outbreak of coronavirus disease 2019 (COVID-19) in December 2019, while approximately 2.9 million people died and 106 million people reportedly recovered as of April 4, 2021 [1]. The initial focus of the healthcare providers, epidemiologists, and policymakers was to identify the epidemiological and clinical features of COVID-19, its management, and prevention strategies as an emerging infectious disease. Medical professionals and researchers consequently concentrated on estimating the disease burden of COVID-19 among the survivors.

Since recovering from COVID-19, several patients complained of persistent or new symptoms [2,3]. Cough, shortness of breath, nausea, headache, palpitations, chest pain, joint pain, physical limitations, vision changes, hearing loss, loss of taste or smell, reduced mobility, numbness in extremities, tremors, myalgia, memory loss, cognitive impairment, mood changes, and others are some of the symptoms reported [4,5]. The terms "long COVID," "post-COVID syndrome," "long haulers," "post-COVID symptoms," and "post-acute COVID-19 syndrome" have been used in the absence of a consensus about how to define these conditions [6].

Several factors have been reported as associated factors for post-COVID symptoms, including increasing age, female sex, presence of comorbidities, psychiatric disorders, obesity, hospital admission, early symptoms, irregular findings in auscultation at symptom onset, and so on [3,7,8]. Younger patients, including those with mild symptoms during the acute phase of infection, are presenting with similar complaints [2,4,9].

Post-COVID symptoms should be identified by health professionals; otherwise, some of the essential features or risk factors for post-COVID symptoms can be ignored, which may carry severe consequences [4]. The current research aimed to estimate the prevalence, length of illness, and risk factors of post-COVID symptoms in order to warn healthcare professionals about the imminent burden of the disease and recognize people at higher risk of suffering.

## Materials and methods

Medical doctors are one of the most vulnerable groups for getting COVID-19 infection. Our study was designed to estimate the prevalence of acute post-COVID symptoms and long post-COVID symptoms among the recovered medical doctors, and find out the risk factors of long post-COVID symptoms.

Study design

This descriptive cross-sectional analysis was carried out among Bangladeshi medical doctors who had been infected with COVID-19 from April 1, 2020, to July 30, 2020. The interval between the participants' COVID-19 detection by a positive reverse transcription-polymerase chain reaction (RT-PCR) for severe acute respiratory syndrome coronavirus 2 (SARS-CoV-2) and the date of data collection was 124.4 (21.6) days on average. The study participants were chosen with the assumption that medical doctors are more knowledgeable of the clinical features of COVID-19 and are more likely to share relevant details than the general public.

Participants in the sample were those who had recovered from acute COVID-19 infection at least two months prior to the date of data collection. Recovery from acute COVID-19 infection was considered when the patient had no fever for three consecutive days without medication or improved in COVID-19 related symptoms or was discharged in case of hospitalization or had two subsequent negative RT-PCR results for SARS-CoV-2, 24 hours apart. The post-acute phase was described as the time period following recovery from acute infection. The post-acute phase was defined as at least two weeks after diagnosis for asymptomatic patients.

Study population and sampling

The Bangladesh Doctors' Foundation, an online philanthropic social network of medical doctors, provided a list of 540 COVID-19 RT-PCR test positive medical doctors with email addresses and mobile phone numbers. Using the formula given by Lwanga and Lemeshow, we estimated the sample size to be 192 based on the unknown prevalence of post-COVID-19 symptoms among all infected patients and a 7% margin of error [10]. After accounting for a 30% non-response rate (from the author's previous survey of medical doctors during the COVID-19 pandemic), our final sample size was 250. Using the Statistical Package for Social Sciences (SPSS) program, samples were drawn randomly from the list of COVID-19 positive medical doctors. The doctors who were unwilling to participate or could not be reached over the phone even after trying for three consecutive days were excluded from the study.

Data collection

The data were collected through telephone interviews over three weeks. For data collection, five resident medical doctors from the Department of Internal Medicine at Bangabandhu Sheikh Mujib Medical University were recruited. A five-day training session was arranged, during which data collectors were taught how to present themselves, explain the purpose of the research, gain informed consent, and collect data using mobile phones. Data collectors scheduled each participant's time in advance, and each interview took about 15 minutes to complete. After receiving approval from the participants, the interviews were audiotaped.

A semi-structured questionnaire was developed to gather information on participants' clinico-demographic characteristics as well as post-COVID symptoms. Age, sex, comorbid conditions, smoking history, hospital admission, intensive care unit (ICU) support, and complication during acute COVID-19 infection were included in the clinico-demographic profile. The smoking history was categorized as never smoker, past smoker (who quit smoking six months ago), and current smoker. Past smokers and never smokers were considered non-smoker during analysis.

The self-reported data on comorbidity were obtained regarding non-communicable diseases (NCDs) like hypertension, diabetes, asthma, thyroid disease, ischemic heart disease, chronic kidney diseases, and chronic liver disease. The participants who reported laboratory-confirmed bacterial coinfection, acute respiratory distress syndrome (ARDS), acute cardiac injury, acute kidney injury, multiorgan dysfunction, sepsis, or shock during the acute phase of COVID-19 illness were considered as having associated complications.

The post-COVID symptoms were defined as the presence of signs and symptoms consistent with COVID-19 during the post-acute phase. Participants were asked whether they had post-COVID symptoms including trouble breathing, cough, chest pain, palpitation, nausea, sleep disruption, memory lapses, lack of concentration, anosmia, loss of taste, loss of appetite, weight loss, muscle pain, joint pain, hair fall, and rash. Participants were also asked how long they had experienced any of these symptoms. We classified the post-COVID symptoms based on their duration. Symptoms persisting ≤60 days following recovery were considered as acute post-COVID symptoms and >60 days following recovery were considered as long post-COVID symptoms.

Data analysis

As summary measures for the categorical variables, frequencies and percentages were measured. Arithmetic means (standard deviation) were used to characterize the numerical results.

A logistic regression model was used to evaluate the relationship between any long post-COVID symptoms and explanatory variables. The variables with a p-value of 0.05 or less in the bivariate model or variables with a significant impact on outcome variables defined in empirical studies were chosen for further analysis in the logistic regression model to classify independent predictors of the outcome variable. The coefficient of inter-correlation with all of the other variables was found to be sufficient (<0.235). The odds ratios (OR) were presented along with the corresponding 95% confidence interval (CI). Data were analyzed using SPSS version 21 (Armonk, NY: IBM Corp.) and the p-value was considered significant at 5% level.

Ethical consideration

Ethical clearance was obtained from the Institutional Review Board of Bangabandhu Sheikh Mujib Medical University (BSMMU/2020/6317). Informed verbal consent was obtained from each study participant after explaining the study procedure, benefit and risk of participation, right to refuse to participate or withdraw from the study, confidential handling of data, and principal investigator's identity. The participants were at liberty of not responding to any question and withdraw from the research at any point.

## Results

A total of 186 medical doctors were interviewed, with a 74.4% response rate.

Clinico-demographic characteristics

The participants' age ranged from 24 years to 75 years with a mean (SD) age of 34.8 (9.9) years. Approximately one-third of participants had at least one comorbid condition, with asthma (19.4 %) and hypertension (14.5%) being the most commonly recorded. Approximately 20% of participants needed hospitalization, and 1.6% required ICU transfer. The incidence of at least one, two, and three or more post-COVID symptoms were 69.9%, 39.8%, and 23.1 %, respectively (Table 1).

**Table 1:** Clinico-demographic features of the participants (N=186). ^a^Non-smoker = Past smoker + Never smoker ICU: intensive care unit; COVID: coronavirus disease

Variables	Frequency	Percentage
Age (in completed years)		
< 50	168	90.3
50 to 59	10	5.4
≥60	8	4.3
Sex		
Male	123	66.1
Female	63	33.9
Comorbidity		
None	117	62.9
Asthma	36	19.4
Hypertension	27	14.5
Diabetes	14	7.5
Thyroid disease	6	3.2
Ischemic heart disease	2	1.1
Smoking status		
Non-smoker^a^	152	81.7
Current smoker	25	13.4
Complication during acute phase	13	7.0
Required hospital admission	38	20.4
Required ICU support	3	1.6
Post-COVID symptoms		
At least one symptom	130	69.9
At least two symptoms	74	39.8
Three or more symptoms	43	23.1

Post-COVID symptoms and duration

The participants reported that most of their post-COVID symptoms relieved within 30 days during the post-acute phase. However, few symptoms like fatigue (8.1%), difficulty in breathing (6.5%), lack of concentration (4.8%), hair fall (4.3%), memory lapses (4.3%), sleep disturbance (3.8%), and joint pain (1.6%) persisted >60 days even after recovery (Table 2).

**Table 2:** Duration of post-COVID symptoms (N=186). ^a^Percentage calculated for total participants. COVID: coronavirus disease

Post-COVID symptoms	Duration during the post-acute phase			
	Frequency (percentage)^a^ of participants			
	Total N=186	Acute post-COVID symptoms		Long post-COVID symptoms
		<30 days	31-60 days	> 60 days
Cardio-respiratory				
Difficulty in breathing	19 (10.2)	3 (1.6)	4 (2.2)	12 (6.5)
Cough	12 (6.5)	10 (5.4)	2 (1.1)	-
Palpitation	11(5.9)	9 (4.8)	2 (1.1)	-
Chest pain	5 (2.7)	3 (1.6)	1 (0.5)	1 (0.5)
Rhinorrhea	2 (1.1)	2 (1.1)	-	-
Sore throat	1 (0.5)	1 (0.5)	-	-
Neuro-psychiatric				
Fatigue	80 (43.0)	55 (29.6)	10 (5.4)	15 (8.1)
Sleep disturbance	25 (13.4)	17 (9.2)	1 (0.5)	7 (3.8)
Lack of concentration	22 (11.8)	10 (5.4)	3 (1.6)	9 (4.8)
Memory lapses	13 (7.0)	4 (2.2)	1 (0.5)	8 (4.3)
Headache	12 (6.5)	8 (4.3)	3 (1.6)	1 (0.5)
Anosmia	11(5.9)	7 (3.8)	4 (2.2)	-
Irritability	8 (4.3)	6 (3.2)	-	2 (1.1)
Loss of taste	6 (3.2)	4 (2.2)	1 (0.5)	1 (0.5)
Depressed mood	3 (2.6)	3 (2.6)	-	-
Anxiety	3 (1.6)	2 (1.1)	-	1 (0.5)
Vertigo	2 (1.1)	2 (1.1)	-	-
Gastrointestinal				
Loss of appetite	7 (3.8)	6 (3.2)	-	1 (0.5)
Weight loss	4 (2.2)	4 (2.2)	-	-
Diarrhea	3 (1.6)	3 (1.6)	-	-
Abdominal pain	1(0.5)	1 (0.5)	-	-
Nausea	1 (0.5)	-	1 (0.5)	-
Musculoskeletal				
Muscle pain	12 (6.5)	12 (6.4)	-	-
Joint pain	8 (4.3)	5 (2.7)	-	3 (1.6)
Cutaneous				
Hair fall	9 (4.8)	1 (0.5)	-	8 (4.3)
Rash	2(1.1)	2 (1.1)	-	-

Risk factors of long post-COVID symptoms

Among all the participants, 44 (23.7%) reported suffering from at least one long post-COVID symptom. The adjusted logistic regression model for the persistence of any long post-COVID symptoms showed that female participants are 2.79 times more likely to suffer from long post-COVID symptoms than their male counterparts (OR, 2.79; 95% CI, 1.28-6.06; p-value: 0.010). Participants with comorbid conditions are 2.28 times more likely to have persistent post-acute symptoms than others (OR, 2.28; 95% CI, 1.08-4.79; p: value, 0.030) (Table 3).

**Table 3:** Risk factors for the long post-COVID symptom (N=186). ^a^All of the risk factors listed in the table were simultaneously adjusted using logistic regression, with the persistence of at least one long post-COVID symptom. ^b^p-Value < 0.05. ^c^p-Value <0.01. ^d^Participant with any comorbid condition. COVID: coronavirus disease

Variables	Presence of long post-COVID symptom	
	Unadjusted odds	Adjusted odds^a^
Age	1.03 (0.99-1.06)	1.03 (0.99-1.07)
Sex		
Male	Referent	Referent
Female	2.46 (1.23-4.93)^b^	2.79 (1.28-6.06)^b^
Comorbidity		
Absent	Referent	Referent
Present^d^	2.58 (1.29-5.16)^c^	2.28 (1.08-4.79)^b^
Smoking status		
Non-smoker	Referent	Referent
Current smoker	0.39 (0.11-1.40)	0.62 (0.16-2.34)
Complication during acute phase		
No	Referent	Referent
Yes	2.15 (0.66-6.94)	2.10 (0.607-7.281)
Hospital admission		
No	Referent	Referent
Yes	1.19 (0.53-2.71)	1.04 (0.42-2.59)

## Discussion

Our study found that 70% of participants had post-COVID symptoms, where about 24% of the participants had at least one symptom for more than 60 days even after recovering from acute illness. Several recent studies reported diverse pictures of post-COVID symptoms with different durations. In a study from Italy, Carfì et al. found 87% of hospitalized COVID-19 patients had persisting symptoms related to COVID-19, where 43% had one or two symptoms, and 55% had three or more symptoms after an average of 60 days of their initial symptoms [11]. Similarly, Carvalho-Schneider et al. reported that 66% of their patients had long-COVID symptoms even after 60 days [8]. Tenforde et al. reported that about 35% of COVID-19 infected patients did not return to usual health after two to three weeks of their diagnosis in a multi-country study [7]. Apart from methodological variation, the absence of a uniform definition for the acute and post-acute phases makes the data incomparable across the world. Setting a post-COVID timeline will help determine whether the features are local or universal [12].

The findings of this study showed that the frequency of post-COVID symptoms decreased over time, as reported by other follow-up studies on COVID-19 [7,8]. This information is helpful for patient management. However, the duration and severity might vary based on the clinical and demographic characteristics of patients. These findings need further evaluation by close monitoring of the patients through follow-up studies. Several studies on other coronaviruses like severe acute respiratory syndrome (SARS) or Middle East respiratory syndrome (MERS) infection showed respiratory dysfunction, reduced exercise capacity, psychological problems such as post-traumatic stress disorder (PTSD), depression, and anxiety, and reduced quality of life beyond six months [13]. Wu et al. found that pulmonary fibrosis in CT findings persisted up to seven years post-infection with SARS [14]. Another study by Lam et al. revealed that 40% of people recovering from SARS had chronic fatigue symptoms 3.5 years after being diagnosed [15].

The most commonly reported post-COVID symptoms in the present study were fatigue, sleep disturbance, lack of concentration, breathing difficulty, memory lapse, myalgia, cough, etc. Carfì et al. found a high proportion of COVID-19 survivors suffered from fatigue (53%) followed by dyspnea (43%), joint pain (27%), and chest pain (22%) [11]. Anosmia/ageusia (59%), dyspnea (30%), or asthenia (40%) were reported as common symptoms by Carvalho-Schneider et al. [8]. Garrigues et al. reported continued symptoms like fatigue (55%), dyspnea (42%), memory loss (34%), sleep disorders (31%), and difficulty with concentration (28%) among recovered patients after three months of diagnosis [16]. With varied duration and frequency, these studies, however, reported an almost similar type of symptoms.

Our study findings suggest that females and participants with comorbid conditions are more likely to suffer from long post-COVID symptoms. Few other studies have also shown that females are at a higher risk of being a long hauler [3,17]. Tenforde et al. reported that having chronic medical conditions is a risk factor for persisting symptoms of COVID-19 [7].

There were some shortcomings in this report. Since this was a cross-sectional study rather than a follow-up study, there is a risk of recall bias. Furthermore, we could not determine whether post-COVID symptoms were chronic or newly formed acute phase symptoms. As the telephone interview method was used, the nonrespondents may also have varied from survey participants.

## Conclusions

This study attempted to quantify the problem of post-COVID symptoms to prepare the healthcare providers for the forthcoming burden. Female sex and comorbid disease conditions are found as risk factors for long post-COVID symptoms. As most patients showed declining symptoms over time, counseling the patient regarding illness will set expectations. However, patients with incapacitating symptoms should be monitored closely. Pragmatic rehabilitation and psychiatric care programs need to be developed based on the prospective research findings.
